# Supplementary material for: Metal binding site alignment enables network-driven discovery of recurrent geometries across sequence-divergent proteins and drug off-targets
Source: PLoS Comput Biol. 2026 Aug 28;22(8):e1014636. doi: 10.1371/journal.pcbi.1014636 (PMC13524349; doi:10.1371/journal.pcbi.1014636)
Supplement: Supplementary file 1 [file pcbi.1014636.s001.pdf]

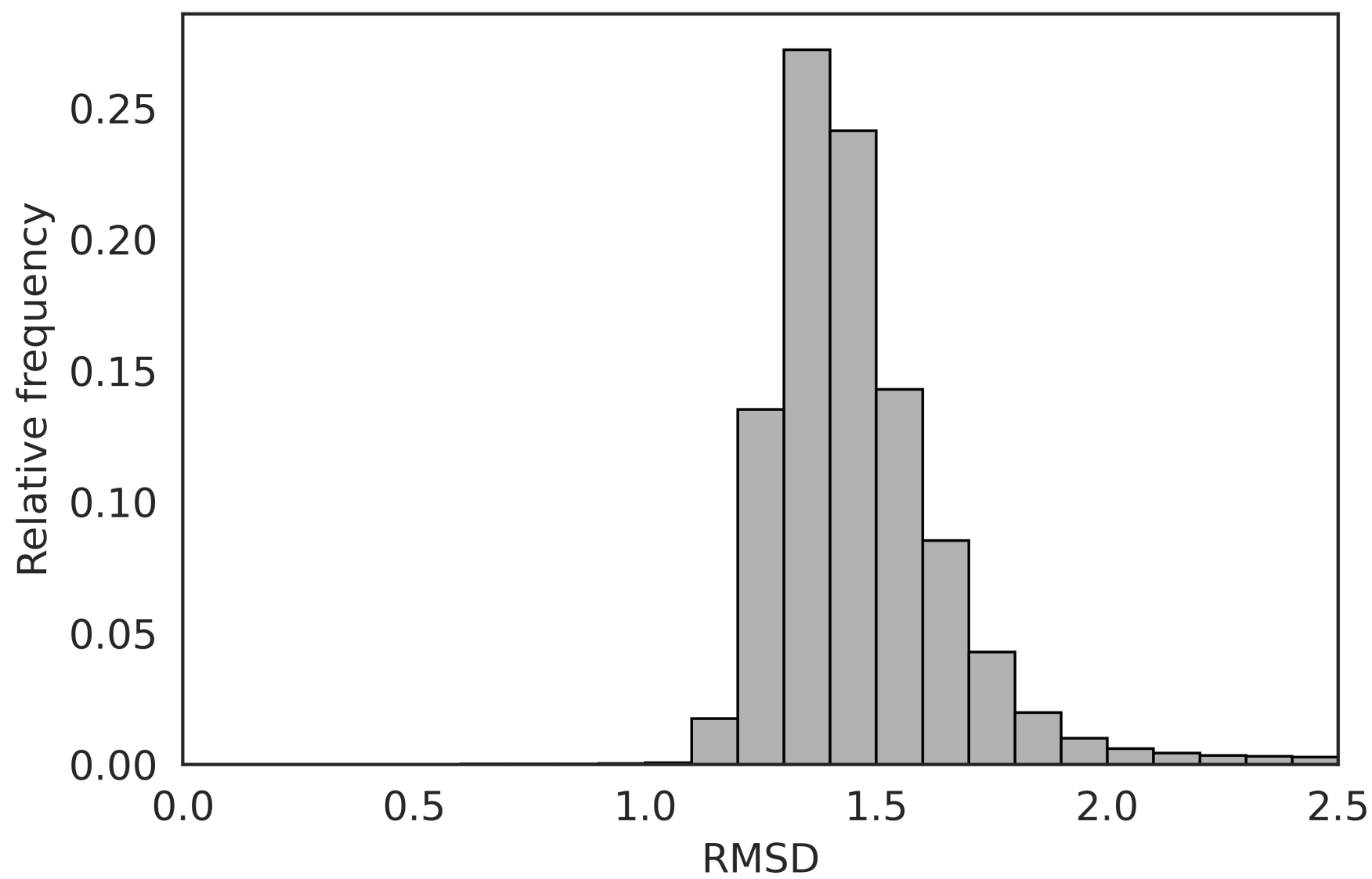

Fig A: Distribution of RMSD values computed for 10^5 randomly selected MBS point cloud pairs, providing a baseline which characterizes the expected RMSD distribution when aligning unrelated structures.

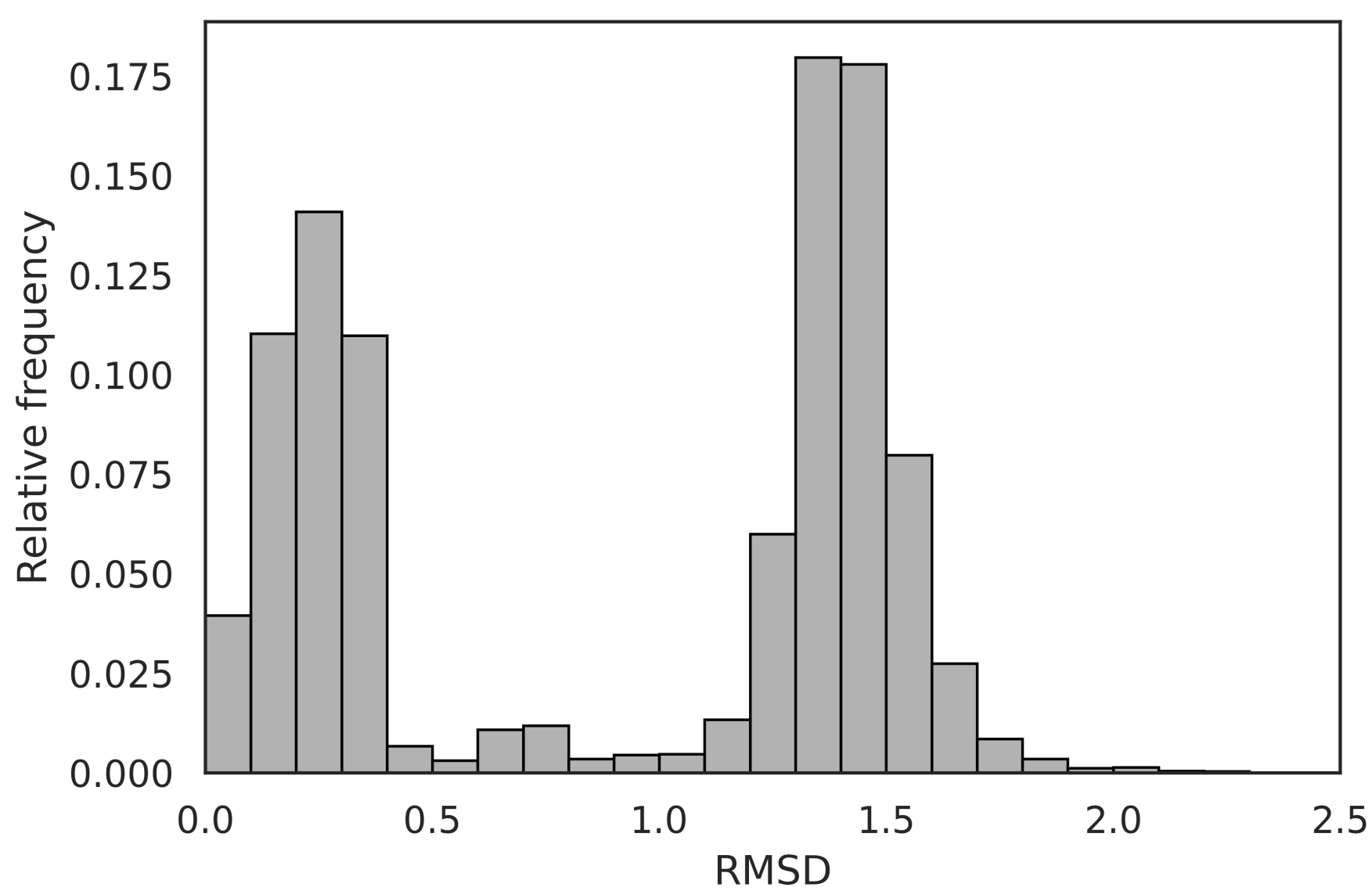

Fig B: Distribution of RMSD values for ICP alignment of 10^4 pairs of structurally similar MBS point clouds (global-optimum RMSD < 0.4 Å). Initializations were generated using Fast Global Registration, followed by fine-tuning using ICP.

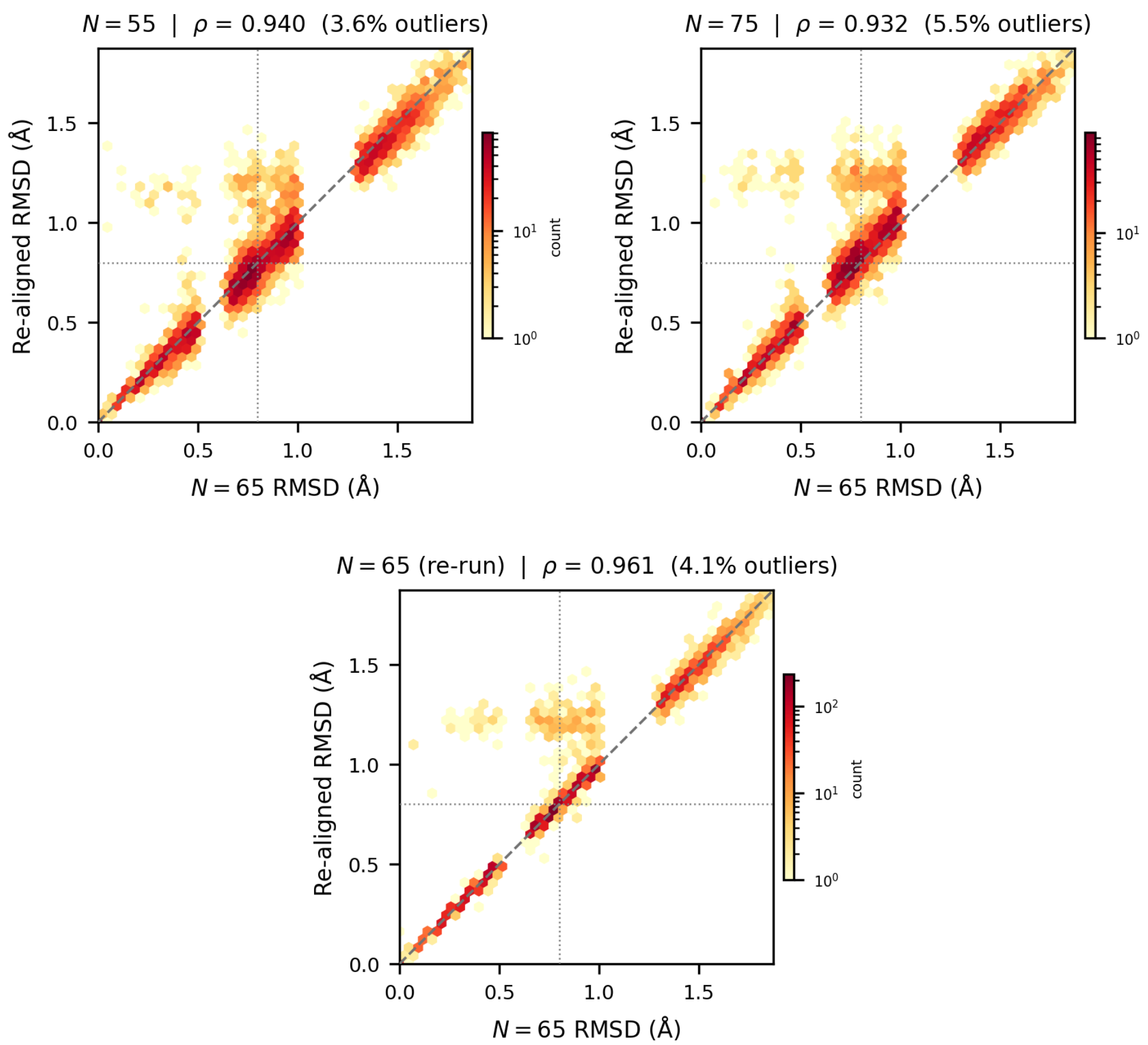

Fig C: Re-aligned RMSD at $N = 55$ (top left), $N = 65$ re-run (bottom centre, ICP stochasticity baseline), and $N = 75$ (top right) versus the stored $N = 65$ RMSD. Colour encodes pair density on a log scale. The dashed line denotes the identity ($y = x$); dotted lines mark the classification threshold at 0.8 Å. The analysis covers MBS pairs drawn from four RMSD strata (10^3 pairs each): clearly positive (RMSD < 0.50 Å), near-threshold positive (0.65–0.80 Å), near-threshold negative (0.80–1.00 Å), and clearly negative (1.30–2.00 Å).

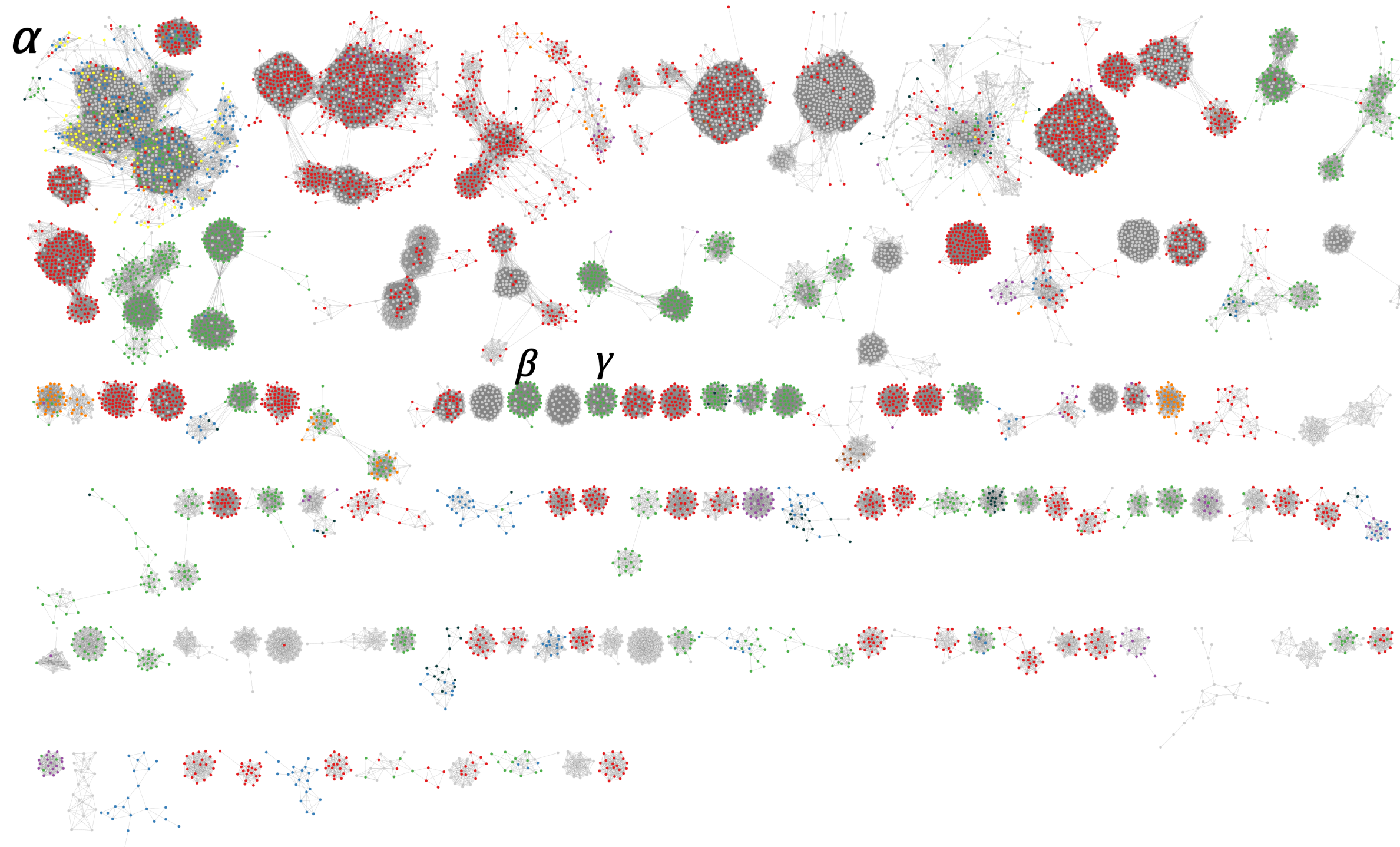

Fig D: Connected components of size $N \geq 20$ of the MBS network. The nodes are colored according to the highest classification digit of their Enzyme Commission (EC) number. Oxidoreductases (EC 1, red), transferases (EC 2, blue), hydrolases (EC 3, green), lyases (EC 4, purple), isomerases (EC 5, orange), ligases (EC 6, yellow), translocases (EC 7, brown). Nodes with ambiguous or undefined EC assignment are colored black and grey, respectively.

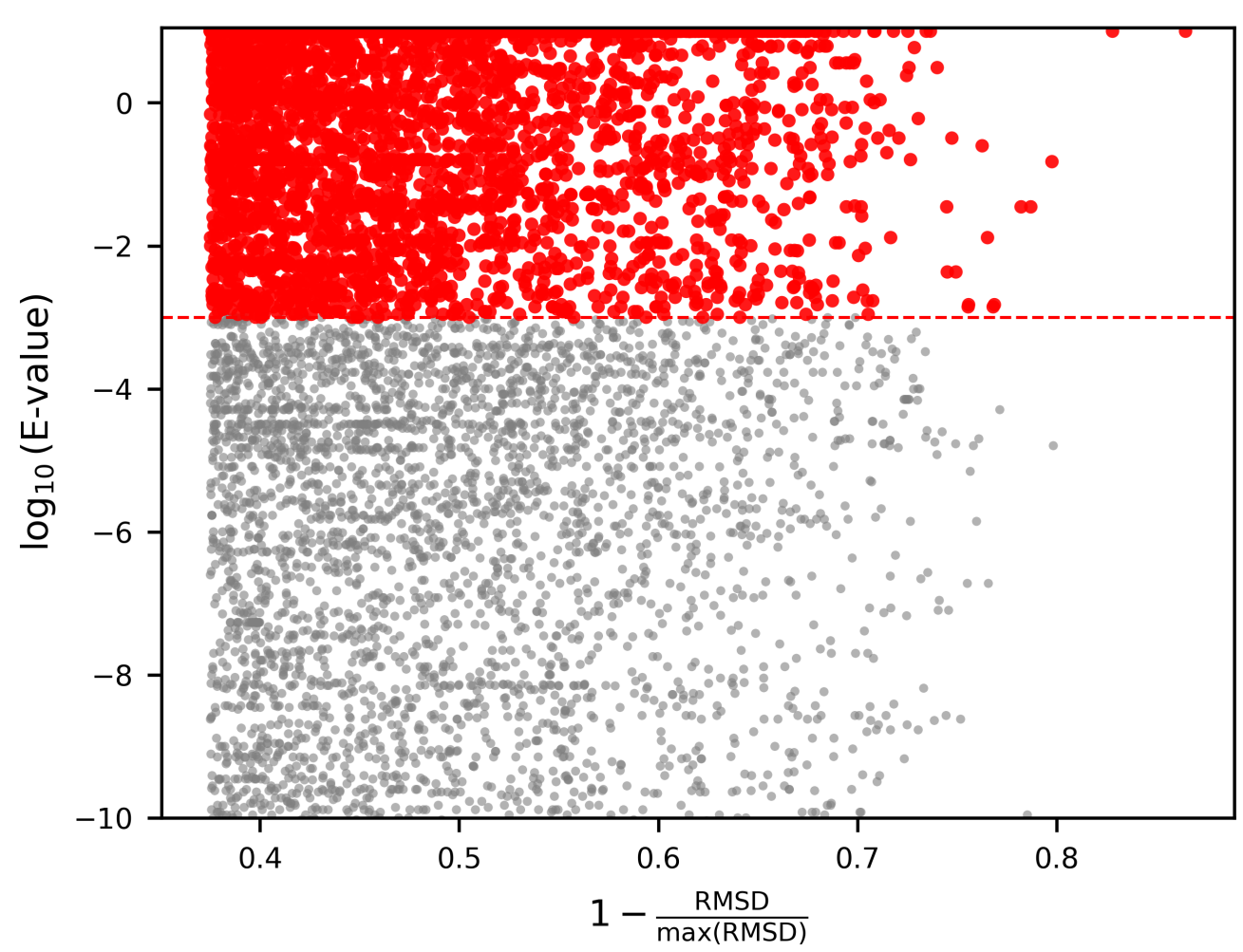

Fig E: Local sequence similarity filtering for pairs in the red dashed box in Fig 10A. The $\log_{10}$(E-value) is obtained from shuffled-sequence Smith–Waterman alignments of the corresponding full-length proteins. The horizontal dashed line marks the threshold ($E < 10^{-3}$) used to distinguish pairs exhibiting statistically significant local sequence similarity (grey points) from those without detectable local similarity (red points).

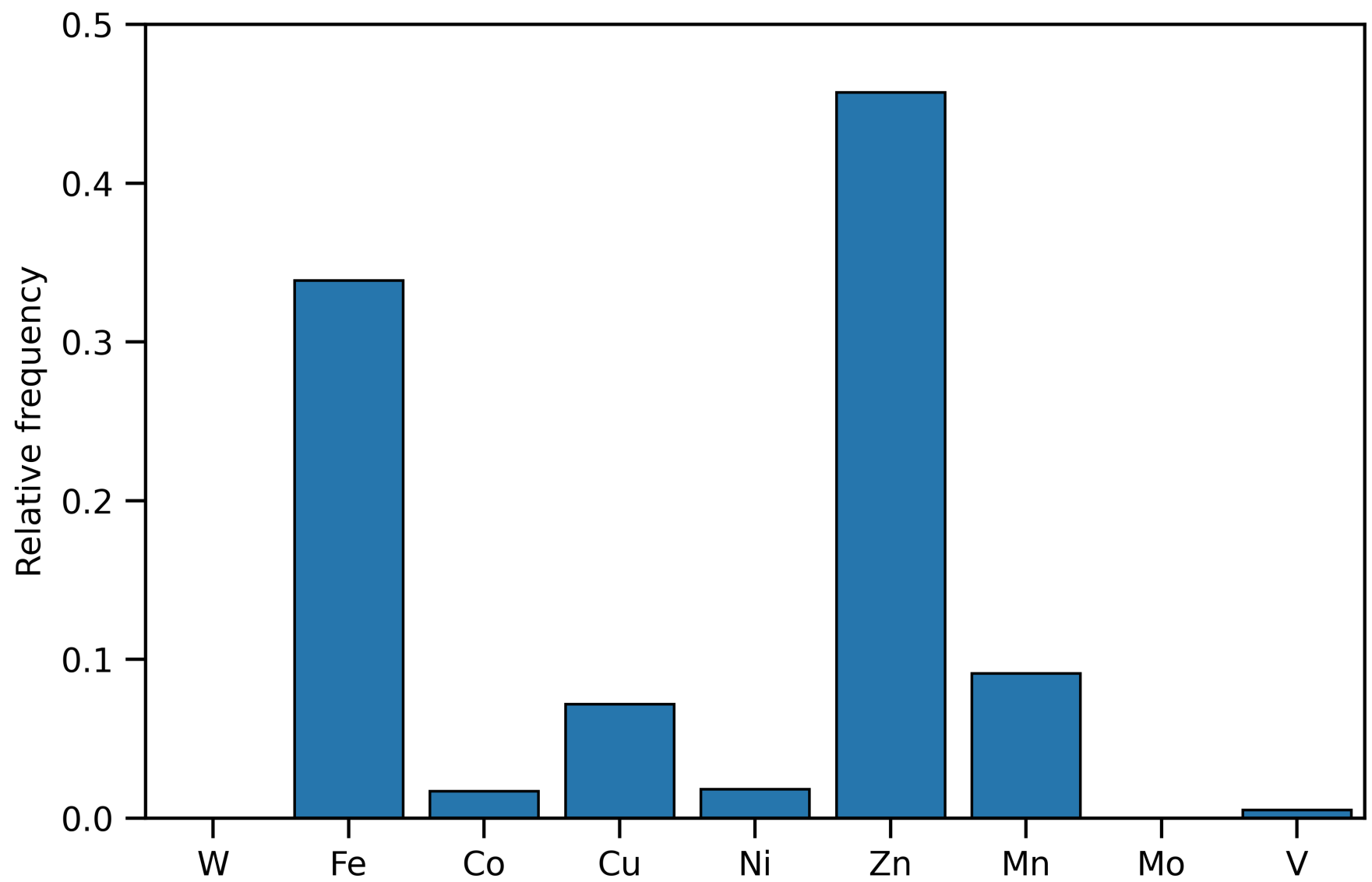

Fig F: Relative distribution of metal ligands of the set of identified MBS pairs with highly similar point cloud geometries across sequence-divergent protein contexts.
